# Single photon superradiance enhanced light–matter interaction in spatially ordered shape and volume controlled single quantum dots: enabling on-chip photonic networks

**DOI:** 10.1515/nanoph-2025-0270

**Published:** 2025-09-10

**Authors:** Lucas Jordao, Swarnabha Chattaraj, Qi Huang, Siyuan Lu, Jiefei Zhang, Anupam Madhukar

**Affiliations:** Nanostructure Materials and Devices Laboratory, 5116 University of Southern California, Los Angeles, CA 90089-0241, USA

**Keywords:** single photon sources, superradiance, ordered quantum dots, quantum information processing, molecular beam epitaxy, scanning transmission electron microscopy

## Abstract

On-chip photonic networks require adequately spatially ordered matter-photon interconversion qubit sources with emission figures-of-merit exceeding the requirements that would enable the desired functional response of the network. The mesa-top single quantum dots (MTSQDs) have recently been demonstrated to meet these requirements. The substrate-encoded size-reducing epitaxy (SESRE) approach underpinning the realization of these unique quantum emitters allows control on the shape, size, and strain (lattice-matched or mismatched) of these epitaxial single quantum dots. We have exploited this unique feature of the MTSQDs to reproducibly create arrays of quantum dots that exhibit single photon superradiance, a characteristic of the SESRE-enabled delicate balance between the confinement potential volume, depth, the resulting exciton binding energy, and the degree of confinement of the center of mass (CM) motion of the exciton. Scanning transmission electron microscope (STEM) studies reveal the structural (atomic scale) and chemical (nanometer scale) nature of the material region defining the notion of the shape and volume (here large) of the electron confinement region (*i.e.* the QD). In the exciton’s weak CM confinement regime, owing to its coherent sampling of the large volume, an enhancement of the MTSQD oscillator strength to ∼30 is demonstrated. Theoretical modelling with input from the STEM findings provides corroboration for single photon superradiance causing enhancement of the oscillator strength by ∼2.5–3. Our findings allow fabricating and studying interconnected networks enabled by these unique matter qubit-light qubit interconversion units that can be realized for lattice matched and mismatched material combinations covering UV to mid-infrared wavelength range.

## 1 Introduction

Conversion of information from matter qubit to light qubit back and forth with the highest fidelity is at the core of all quantum information processing (QIP) hardware approaches [1], [2], [3]. This conversion, in turn, is controlled by light–matter interaction. Thus, implementation systems and approaches that can tailor light–matter coupling are of considerable significance to QIP systems employing any of the major exploited physical hardware platforms: matter qubits represented in atoms, ions, structural and/or chemical defects in solids, semiconductor quantum dots, and Josephson junction based superconducting circuits. The conceptual and operational physics of matter-photon qubit conversion in these platforms has usually been modelled as an electric dipole driven transition in an effective two-level matter system. It has guided the interpretation of the transition rate ( $T_{1}^{-1}$ ) as the product of the transition oscillator strength, $f\left(\omega \right)$ , and the available local density of photon states at the transition frequency (*ω*), $\rho \left(r_{0},\omega \right)$ [4].

(1) $$\begin{array}{c} T_{1}^{-1}\propto f\left(\omega \right)\times \rho \left(r_{0},\omega \right) \end{array}$$

For most matter qubits, such as created in atoms, ions and solid-state emitters (defects/deep levels) the two-level transition oscillator strength *f* is essentially fixed [5], [6], [7] and the transition (decay) rate is manipulated primarily by tailoring the local photon density of states, $\rho \left(r_{0},\omega \right)$ . This is achieved through modification of the dielectric environment around the emitter by such means as embedding the emitter in a cavity and/or waveguide designed to *enhance* the local density of photon states to which photons couple, thereby enhancing the matter qubit (typically exciton) decay rate. By contrast, semiconductor quantum dots (QDs) are a unique class of quantum emitters in which the oscillator strength for exciton decay itself can be manipulated (enhanced) through control on the relative volume and strength (depth) of the confinement potential, and the resulting binding energy and volume of the exciton formed by the excitation of the electron from the confined highest valence band derived state to the lowest confined conduction band derived electron state [8]. This is because of the *single photon* superradiance effect [9], [10] which, amongst the inorganic quantum emitters under investigation for quantum information, is realizable only in QDs as these can be tailored to exhibit weak confinement of the exciton’s center-of-mass in a volume larger than its own, leading to enhancement of the atomic oscillator strength arising from energy storage in a *coherent* collective quantum state shared across the atoms of the confining volume of the dot. The enhanced oscillator strength, *f*, in turn gives enhanced light–matter interaction.

Strong light–matter interaction is particularly important to developing multiple emitter-based quantum networks as network system-level performance imposes strict requirements on the characteristics of the individual quantum emitters constituting the platform to be employed [11]. As we discussed in ref. [11], the individual emitter’s single photon characteristics must consist of near unity quantum efficiency, single photon purity, and indistinguishability in order for it to meet the requirements for QIP applications such as linear optical quantum computing (LOQC) and Boson sampling [12], [13]. Beyond these individual characteristics, interconnecting emitters to realize system-level quantum circuits/networks for QIP applications demands: (1) designed on-chip positioning of the emitters to nanometer accuracy for optical wavelength regime, and (2) emission wavelength nonuniformity of the emitters within the threshold allowed for on-chip tuning technologies (e.g. ∼1 V–3 V applied bias for a ∼3 nm wavelength shift via the Stark effect in quantum dots) [14], [15] thereby enabling multi-photon interference. Strong light–matter interaction, albeit not a strict requirement for such platforms, is highly beneficial as the resultant faster radiative decay lifetime of the emitters would lead to increased robustness to intrinsic dephasing [16]. This would also allow the system to operate at higher frequencies [17], [18].

**Figure 1:**
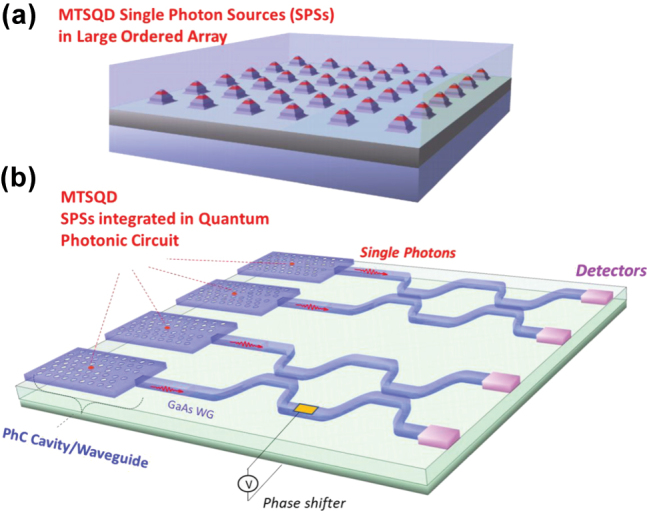
Shows symbolically the single quantum dot (red regions) synthesized selectively on top of *in situ* created quantum scale mesa tops in designed spatially ordered arrays (panel a). Upon completion of synthesis, the guiding nanoscale patterned mesa morphology has been planarized, realizing the platform for subsequent fabrication of interconnected network of quantum emitters as symbolically indicated (panel b) by the QDs surrounded by photonic crystal cavity/waveguide seamlessly connecting to ridge waveguides that enable horizontal emission and propagation of photons as required for on-chip quantum photonic circuits.

To date the main limitation in achieving aforementioned platform with quantum dot-based emitters has come from the lack of *adequate* control over the QD positions and their size, shape and composition (*i.e.* the effective 3D confinement potential) across the grown sample due to the random nature of the process by which the most popular employed epitaxial QDs are synthesized – the lattice-mismatched strain-driven self-assembled quantum dots (SAQDs) [19], [20] and the droplet epitaxy quantum dots (DEQDs) [21], [22]. For SAQDs and DEQDs, the lack of adequate spatial positioning precludes developing on-chip quantum optical circuits and the lack of adequate control on size, shape, and volume prevents exploiting the benefits of enhanced light–matter interaction arising from superradiance made possible by the mesoscopic nature of QDs. These limitations have been overcome by a new class of quantum dots dubbed mesa-top single quantum dots (MTSQDs) that we have developed [11] and it is the aim of this paper to report the additional controlled incorporation of the single photon superradiance effect in MTSQDs synthesized in scalable spatially ordered arrays. To aid the discussion of the structural and chemical nature of these MTSQDs that enable generation of their remarkable single photon characteristics, Figure 1 captures symbolically their essence.

In this paper we present scanning transmission electron microscopy (STEM) and energy dispersive X-ray spectroscopy (EDS) based structural/compositional findings on this unique class of QDs, the MTSQDs, that are not only synthesized in adequately accurate controlled locations, with spectral emission characteristics that satisfy all individual- and system-level requirements for QIP [11], but which can also be reproducibly synthesized to show controlled, large oscillator strengths arising from single-photon superradiance. Our results on the control over MTSQD positioning, size, shape, volume, composition and thus the resulting confinement potential depth and profile (across the typically ill-defined heterojunction interface), enabled by the *substrate encoded size reducing epitaxy* (SESRE) [23], [24], [25] growth approach employed, allow for reproducibly synthesizing scalable arrays of quantum emitters with enhanced light–matter interaction. Indeed, neutral exciton radiative decay lifetimes *T*_1_ < 400 ps and large oscillator strengths (*f* ∼ 30) are demonstrated for large arrays. Moreover, MTSQD’s fast radiative decay rates allow for remarkable robustness to intrinsic phonon-dephasing which has led to the demonstration of high single photon indistinguishability (∼94 %) and high 2-qubit CNOT gate operation fidelity (∼90 %) [26]. These results underpin MTSQD’s high promise as technologically relevant on-chip platform for QIP.

## 2 Results and discussion

### 2.1 Spatially ordered arrays of large-volume shape-controlled superradiant MTSQDs

**Figure 2:**
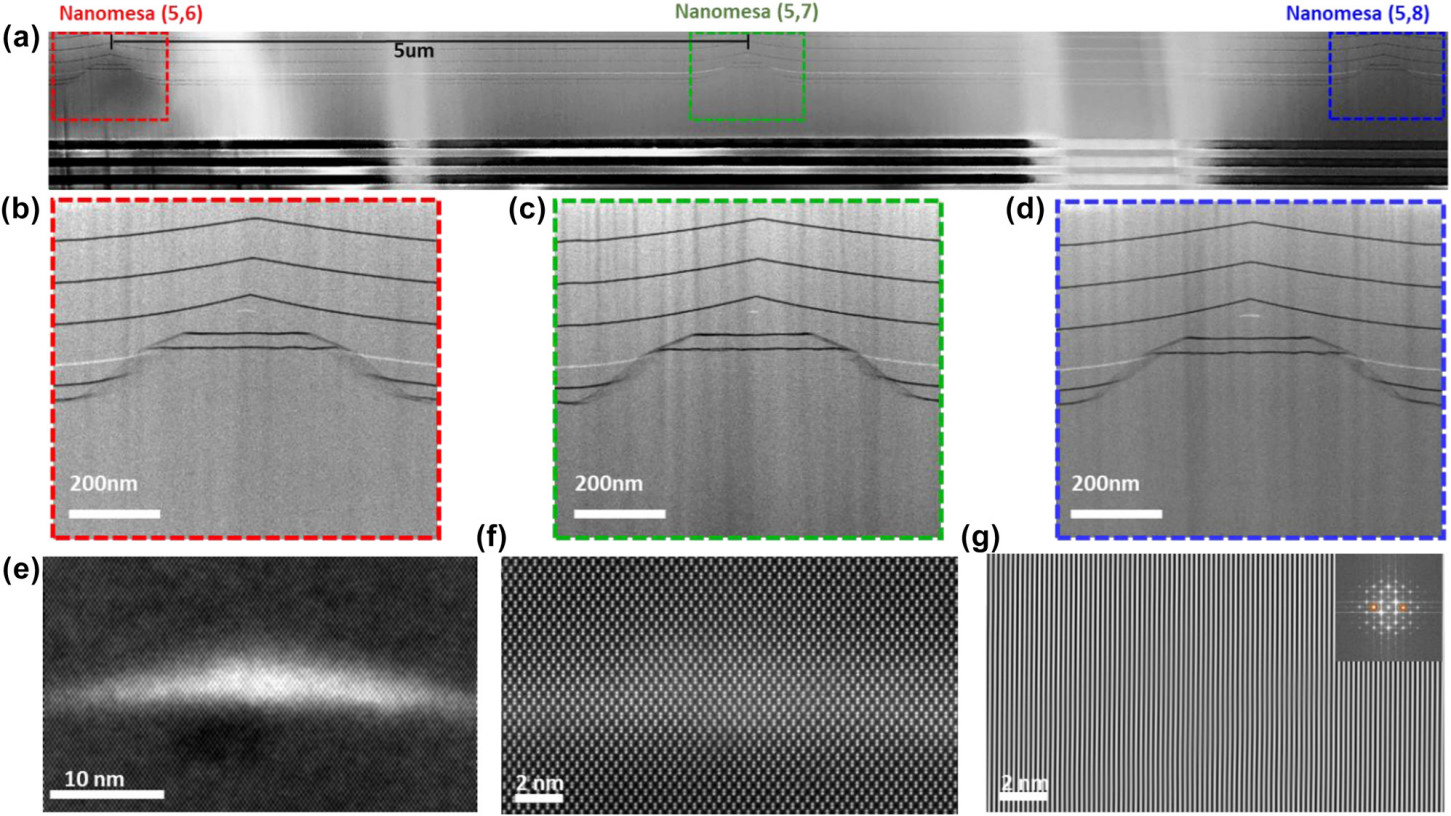
Structural characterization of spatially ordered arrays of large-volume and shape-controlled MTSQDs in a (5 × 8) array. (a) Low-magnification [110] cross-sectional Z-contrast STEM image (top panel) of a TEM lamella specimen with three nanomesas, each containing a MTSQD, from an array. (b)–(d) Z-contrast STEM images of the nanomesas in (a) revealing the growth front evolution during SESRE growth and the MTSQDs at the apex of the nanomesas. (e) Z-contrast STEM image (with Gaussian Blur filter) of MTSQD (5,6) showing its large-volume and unique shape. (f) Atomic-resolution HAADF STEM image of defect-free MTSQD (5,6). (g) Fourier/Bragg filtered image of (f) using the {220} “Bragg” spots (inset shows the FFT of (f), with orange circles indicating where the mask for the filtering was applied).

The SESRE approach based MTSQDs in arrays were examined for their structure and composition using STEM and EDS at various resolutions, reaching atomic. Results on the structural characterization of large-volume shape-controlled MTSQDs are shown in Figure 2 (details on the sample structure and growth are given in the Experimental Methods section). A low-magnification STEM image of the TEM lamella specimen prepared from a row of MTSQDs in a (5 × 8) array is shown in Figure 2(a), and Z-contrast STEM images of the individual nanomesas, from the lamella specimen of Figure 2(a), are shown in Figure 2(b)–(d). The growth front profile evolution of SESRE growth on the pedestal nanomesas is illustrated in Figure 2(b)–(d), where the dark lines are the AlAs marker layers and the grey contrast is GaAs. As material is deposited on the nanomesas, {103} sidewall facets form and the (001) mesa top lateral size reduces owing to the surface stress gradient-driven preferential migration of cations (Ga and Al) from the sidewall to the top and incorporation of adatoms on the [001] mesa top. Once the mesa top size reduces to ∼30 nm, In_0.5_Ga_0.5_As is deposited to form the MTSQD [24] as revealed by the white contrast at the apex of the nanomesas on the STEM images of Figure 2(b)–(d). The position accuracy of each MTSQD with respect to the nanomesa’s center is ∼3 nm laterally (in the [110] direction) and ∼1 nm vertically (in the [001] direction). This high position accuracy of MTSQDs is due to the control on SESRE growth discussed below where all nanomesas evolve with the same profile during growth resulting in MTSQDs always forming centered at the apex of the nanomesas. The as-patterned positioning of the MTSQDs with respect to each other within the array is 5000 nm, with its accuracy limited by the spatial resolution of electron-beam lithography (EBL) patterning (∼5 nm) and fluctuations in size of the *as-etched* nanomesas in the array.

MTSQDs are grown via the SESRE approach which ensures reproducible control of their size and shape across the spatially-ordered arrays. Briefly, SESRE growth of MTSQDs comprises three stages [27]: (i) nanomesa top size reduction, (ii) QD formation at the mesa top and nanomesa pinch-off, and (iii) surface morphology planarization. All these stages of growth are captured by the STEM images in Figure 2(b)–(d). In stages (i) and (ii) of growth, interfacet migration of adatoms from sidewalls to mesa top and preferential incorporation of adatoms leads to mesa top size reduction, enabling deposition of QD material at a mesa top size appropriate for single QD formation and resulting in the formation of an MTSQD near the apex of the nanomesa before pinch-off. Continued growth allows the nanomesa pyramidal morphology to pinch-off [23], [24] and be subsequently planarized [27] during stage (iii) of growth, as shown by the continuous profile of the AlAs marker layers deposited after the MTSQD. Planarization is achieved due to the nanomesa pedestal morphology, with {101} base facets, which allows for a reversal of adatom migration after nanomesa pinch-off; the shallow {100} vertical sidewalls of the nanomesa provide a contiguous link between the {103} sidewalls and the {101} base facets permitting adatoms to migrate away from the nanomesa sidewalls and towards surrounding planar regions. This adatom migration away from the nanomesa increases the growth rate of the planar region with respect to the nanomesa pyramidal morphology and thus buries the nanomesa [27]. At the end of the growth process, we obtain a structure containing spatially ordered arrays of MTSQDs buried in an epitaxial GaAs layer with flat morphology (symbolized by the red regions in Figure 1(a)). Such structures are suitable for deterministically integrating MTSQD quantum emitters with co-designed emitted-photon manipulating passive structures such as waveguides and photonic cavities (Figure 1(b)), fabricated using state-of-the-art protocols [28]. These are the needed interconnected building blocks for realizing the desired scalable quantum networks.

Figure 2(e) shows a Z-contrast STEM image of the MTSQD on nanomesa (5,6) revealing its large-volume and shape. A Gaussian blur filter was applied to the STEM image to enhance the Z-contrast between the InGaAs MTSQD and the surrounding GaAs, better showcasing the MTSQD’s size and shape. The size and shape of MTSQDs formed during InGaAs deposition is strongly influenced by: (i) nanomesa’s top size and morphology, and (ii) growth conditions employed. From STEM characterization it is observed that the base length of MTSQDs along [110] cross-section is roughly equal to the nanomesa opening top size when the QD material (InGaAs) is deposited. Additionally, we employ reproducible growth conditions in our SESRE growths by using the machine-condition transfer-function approach [29] which ensures that growth conditions, and the resulting nanomesa profile evolution, can be controlled from run-to-run. Therefore, by controlling the nanomesa profile evolution, and thus the mesa top opening size before QD deposition, we can control the size and shape of MTSQDs formed. We note that the MTSQDs reported in this paper are bound by {103} facets, however control of the nanomesa morphology and growth conditions also allows for formation of MTSQDs bound by steeper {101} facets as we reported in references [24], [30], [31]. Such control on shape would allow not only for finer control over the MTSQD’s structural and compositional properties but also enables the vertical coupling of the electronic states of multiple individual MTSQDs on a single mesa [32], [33]. The large-volume and distinct shape of the MTSQD shown in Figure 2(e) is observed consistently across multiple MTSQDs inspected in the arrays. This highlights the ability to control the MTSQD shape, size, and, via composition, the confinement potential depth, a capability unique to SESRE growth that allows tailoring light–matter interaction in the MTSQDs.

An atomic-resolution STEM image of the MTSQD of Figure 2(e) is shown in Figure 2(f). The individual Ga, As and In atomic-columns, with spacing of ∼0.141 nm projected along the [1-10] direction (*i.e.* the electron beam direction), are clearly resolved and show that the MTSQD is defect-free. The atomic columns containing In atoms appear brighter than columns containing only Ga and As atomic columns due to In having a larger atomic number; In atoms have larger elastic scattering cross-sections than Ga and As atoms and so more signal is collected at the high-angle annular dark field (HAADF) detector when the beam scans across regions with In atoms. Furthermore, to further examine for the presence of extended defects such as dislocations or stacking faults, the STEM image from Figure 2(f) was Fourier/Bragg filtered and the result is shown in Figure 2(g). The image in Figure 2(g) was produced using the {220} Bragg spots and shows that the {220} lattice fringes in the MTSQD and the surrounding GaAs are without any discontinuities, evidencing that the MTSQD is defect-free, *i.e.* coherent.

The reproducible nature of MTSQD growth allows us to make inferences about and correlations to their optical behavior, such as the observed large oscillator strengths [34], as discussed next.

### 2.2 Scaling of array size of spectrally uniform shape-controlled large volume MTSQDs

**Figure 3:**
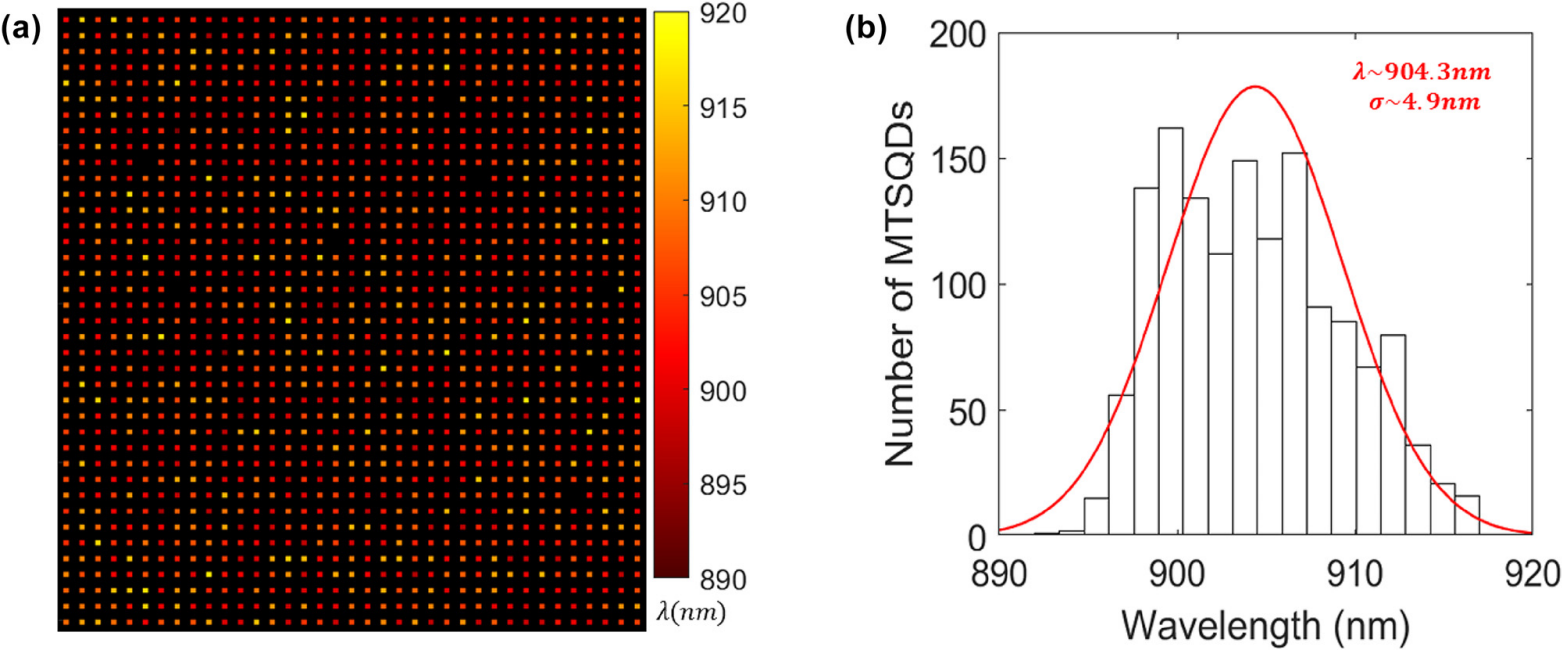
Spectrally uniform scalable arrays of MTSQDs. (a) Color-coded image of the emission wavelengths from ∼1,400 MTSQDs in a (50 × 50) array (MTSQDs separated by a 5 μm pitch). (b) Histogram of the emission wavelengths of (a) indicating mean emission (*λ*) at ∼904.3 nm with a ∼4.9 nm standard deviation (*σ*) as extracted from a Gaussian fit.

Results on the spectral inhomogeneity of scalable arrays of large-volume shape-controlled MTSQDs are shown in Figure 3. Spectrally resolved large area photoluminescence (PL) imaging of ∼1,400 emitters from the (50 × 50) array (a portion of the 2,500 MTSQDs in the array, limited by the excitation area) is shown in Figure 3(a) [35]. Analysis of the wavelength resolved image in Figure 3(a) shows that more than 99 % of the MTSQDs are emitting and that the spectral nonuniformity in the array is low, *σ*_*λ*_ < 5 nm (Figure 3(b)). Beyond the low spectral inhomogeneity of MTSQDs in scalable arrays, they also show remarkable single photon emission characteristics with near-unity quantum yield, short radiative decay lifetimes (*T*_1_ < 400 ps), high single-photon purity (>99.5 %), and high single-photon indistinguishability (∼94 %) [11], [26], [36], comparable to state-of-the-art QD single photon sources [37].

### 2.3 Large volume shape-controlled MTSQDs and large oscillator strengths

**Figure 4:**
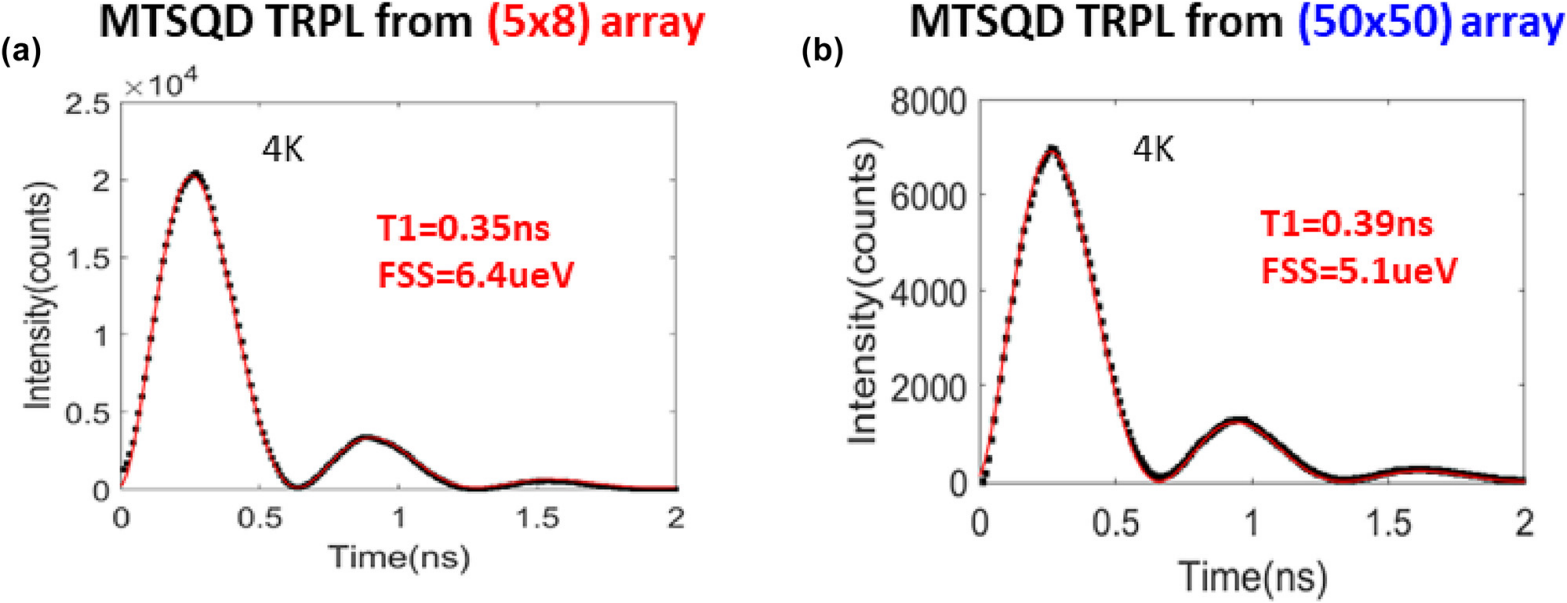
Resonant TRPL from MTSQDs in (5 × 8) and (50 × 50) arrays. (a) Resonant TRPL measurement of a large MTSQD from a (5 × 8) array showing fast decay lifetime (*T*_1_ = 350 ps) and large oscillator strength (*f* ∼ 29). (b) Resonant TRPL measurement of a large MTSQD from a (50 × 50) array (same array as in Figure 3(a)) showing fast decay lifetime (*T*_1_ = 390 ps) and large oscillator strength (*f* ∼ 27).

Time resolved photoluminescence (TRPL) characteristics of the neutral exciton in MTSQDs in the (5 × 8) and (50 × 50) arrays, under resonant excitation, are shown in Figure 4(a) and (b), respectively. The TRPL results are fitted with a three-level system model accounting for fine-structure splitting (FSS) [11] and thus allow us to extract the radiative decay lifetime of the MTSQD’s neutral exciton. MTSQDs from both samples exhibit short decay lifetimes, with *T*_1_ = 350 ps for the MTSQD in the (5 × 8) array and *T*_1_ = 390 ps for the MTSQD in the (50 × 50) array. The oscillator strength of the QD’s neutral exciton within the routinely invoked electric dipole approximation can be determined from its radiative decay lifetime using the formula: $f=\frac{6\pi \varepsilon_{0}m_{0}c^{3}}{nT_{1}F_{p}\omega^{2}e^{2}}$ [38]. The measured *T*_1_ from the MTSQDs in Figure 4 results in *f* ∼ 29 for the MTSQD in the (5 × 8) array and *f* ∼ 27 for the MTSQD in the (50 × 50) array. Note that the oscillation in the emitted single-photon counts in the resonant excitation TRPL measurement is a temporal beat signal, due to the self-interference of the photon wave packet, which comes from the neutral exciton’s FSS [11].

The epitaxial QDs literature, dominated by the lattice-mismatched strain-driven spontaneously formed 3D islands dubbed self-assembled quantum dots (SAQDs) exhibit a typical *T*_1_ of ∼1 ns and an oscillator strength of *f* <10 [39]. By contrast, the MTSQDs reported in Figure 4 exhibit ∼2.5 to 3 times shorter *T*_1_ than SAQDs, indicating oscillator strengths 3 to 4 times larger than SAQDs [11], [40]. Guided by the Z-contrast STEM images exemplified in Figure 2(e) that revealed large confinement volumes (base lengths ∼30 nm and heights ∼5 nm) we inferred that these large oscillator strengths are likely connected not simply to a large confinement volume (Figure 2(e)) but also simultaneously a weak exciton confinement in a sufficiently uniform potential since in other classes of epitaxial quantum dots (Ga-droplet DEQDs, and SAQDs), large confinement volumes alone do not necessarily produce large oscillator strengths [38], [39]. To shed light on the origin of the enhanced oscillator strengths of ∼30 in the MTSQD samples with the (5 × 8) arrays and samples with the scaled up (50 × 50) arrays, synthesized over a year apart, we undertook scanning transmission electron microscope (STEM) based systematic studies of the atomic scale structure and EDS-based nanometer scale spatially resolved distribution of Ga, In and As in our MTSQDs. The findings discussed next corroborate the expectation.

**Figure 5:**
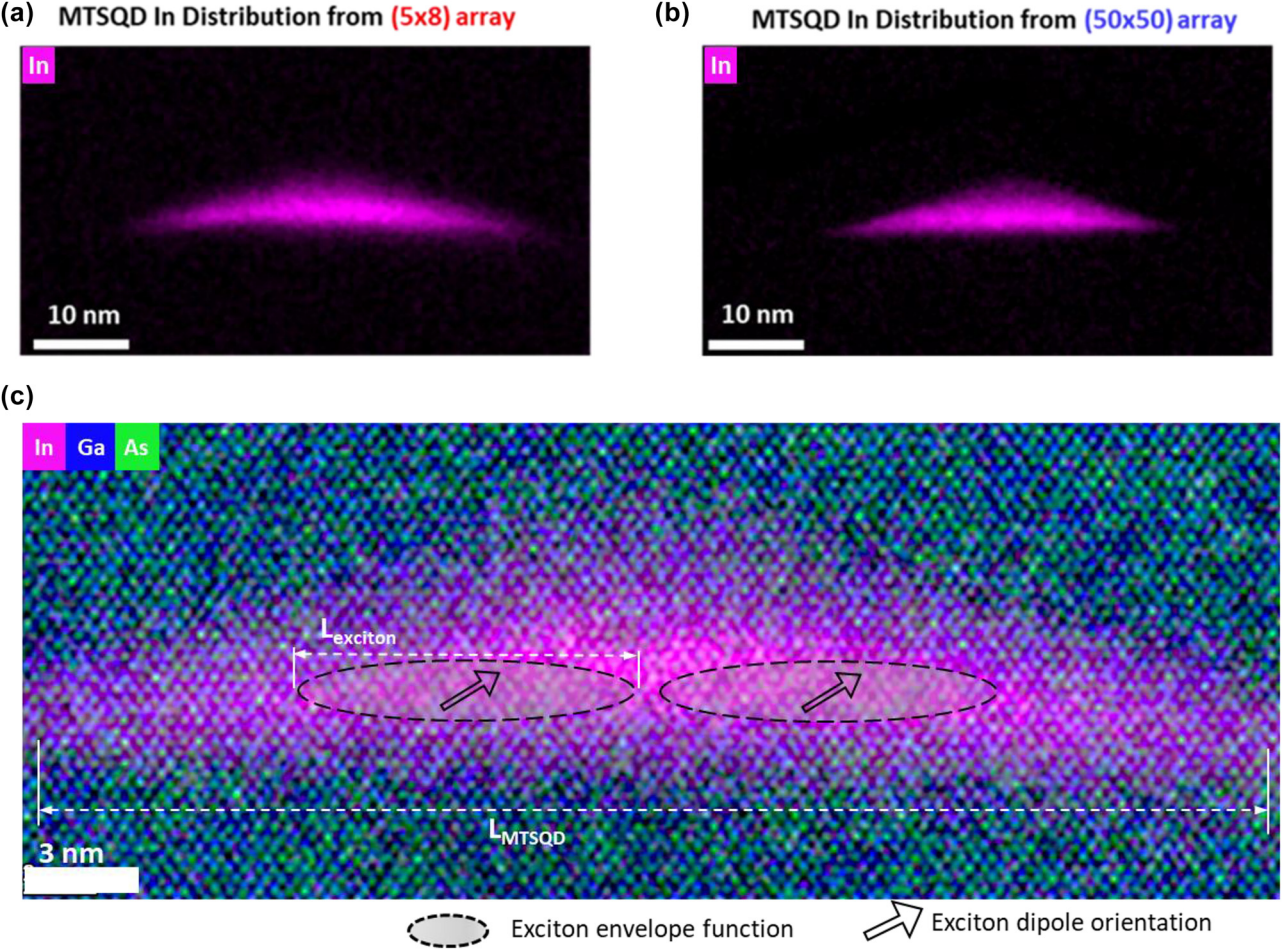
Reproducible large-volume and large oscillator strength of MTSQDs in (5 × 8) and (50 × 50) arrays. (a) Off-zone axis EDS elemental map of a large MTSQD from a small (5 × 8) array, synthesized in the same growth but in a different array from the MTSQD in Figure 3(a). (b) Off-zone axis EDS elemental map of a large MTSQD from a large (50 × 50) array, synthesized in the same growth but in a different array from MTSQD in Figure 3(b). (c) Atomic-resolution EDS elemental mapping of In, Ga, and As distributions in the same MTSQD from Figure 5(a). From this, the height of the MTSQD is seen to be ∼5 nm, and the base length *L*_MTSQD_ ∼30 nm. *L*_MTSQD_ being larger than the exciton Bohr radius (∼10 nm) results in weak-confinement of excitons in MTSQDs (*L*_MTSQD_ > *L*_exciton_) leading to superradiant enhancement of light–matter interaction, as discussed in the text.

Figure 5(a) and (b) show illustrative results from off-zone axis STEM-EDS mapping of In distribution of MTSQDs in both samples ((5 × 8) and (50 × 50) arrays). Both MTSQDs show large volume (with lateral sizes ≥30 nm and heights ≥5 nm) with *uniform* In distribution across the large-volume. A remarkable, yet not surprising, observation from the EDS data is the very similar shape of the In distribution in the MTSQDs from the different samples which comes from the ability to control the MTSQD shape with SESRE growth. In addition to this, we see that the In distribution is uniform across the large base of both MTSQDs which suggests a uniform in-plane confinement potential, a condition necessary for achieving weak-confinement of excitons [4], [10]. Such results are the basis for the observed reproducible large oscillator strength in MTSQDs and the concomitant superradiant enhancement of light–matter interaction as discussed below.

The importance of the uniformity and depth of the confinement potential, beyond the large volume, for producing QDs with excitons in the weak-confinement regime cannot be overemphasized. Quantum dots with small oscillator strengths, *f* < 10, but with large volume have been reported for both GaAs DEQDs (with lateral size >60 nm and height ∼20 nm) [38] and InGaAs SAQDs (with lateral size ∼40 nm) [39]. The small *f* values observed were attributed to the non-uniform confinement potential of the QDs arising from fluctuations in their composition, showing that synthesizing large-volume QDs alone is not enough for producing large oscillator strengths. Some large volume GaAs DEQDs (with lateral sizes ∼40 nm–50 nm and heights ∼5 nm–10 nm) however have shown excitons with short radiative decay lifetimes (*T*_1_ ∼ 200 ps–300 ps) [41], [42], where the observed fast radiative decay rate was inferred to indicate large oscillator strength that is attributed to a speculated low amount of Ga/Al intermixing in the GaAs DEQDs [42]. As demonstrated here with combined STEM and TRPL studies of MTSQDs, achieving excitons with large oscillator strength demands simultaneously (i) a confinement volume significantly larger than the exciton volume, (ii) the exciton motion (i.e. center-of-mass, CM) weakly confined, and (iii) a sufficiently spatially uniform confinement potential that enables coherent motion of the exciton’s CM.

### 2.4 Single-photon superradiance in MTSQDs: enhancement of light–matter interaction

In the concept of superradiance introduced by Dicke [43], a collective enhancement of light emission from an ensemble of *N* emitters occurs when the spatial distance between the emitters (in a given medium) is smaller than the wavelength of the electromagnetic field interacting with the emitters. In this regime, the emitters are coupled through the vacuum modes of the electromagnetic field resulting in a collective excitation of the emitters. The coherent phase relation between the emitters in the ensemble enhances the transition dipole since the excitation can now be localized in any individual emitter of the ensemble, resulting in the emission rate becoming proportional to *N*, the number of emitters in the ensemble. Although this concept was introduced in reference to an ensemble of emitters and has an extensive literature across a variety of physical systems, it is applicable at the level of a single photon and a single QD [10]. Briefly, in QDs, single photon emission arises from excitonic decay of a two-level system determined by the confinement volume of the single quantum dot. In single quantum dots the relevant length scales defining the enhancement of light emission originate in the exciton size (*L*_exciton_) and the confinement potential size (*L*_QD_), where the condition *L*_QD_ > *L*_exciton_ (known as the weak-confinement regime in QDs) allows for the collective sharing of the exciton wavefunction across the *N* unit cells of the QD material, creating a giant transition dipole for the exciton and enhancing the attendant oscillator strength defining the light–matter interaction [9].

The behavior of the exciton in a QD, and thus the strength of light–matter interaction, can be dominated by either electron and hole state quantum-confinement energies in a deep confinement potential (regime of strong quantum confinement of one particle states) or, upon excitation, electron and hole Coulomb attraction effects which are determined by the size of the QD’s confinement potential with respect to the exciton’s Bohr radius [4], [8], [9]. In the absence of Coulomb force, the electron and hole within the QD move independently from each other, and can be characterized by respective envelope functions, $f_{e}\left({\bar{r}}_{e}\right)$ and $f_{h}\left({\bar{r}}_{h}\right)$ . If the quantum confinement potential of electron and hole (determined by the size of the QD) is smaller than the Bohr exciton radius (*a*_*X*_), the electron and hole motion remain decoupled, and this limit is referred to as strong exciton confinement. The result is that the exciton wavefunction becomes delocalized and spreads beyond the confinement volume, with the oscillator strength in the strong-confinement regime being then determined by the overlap of the electron and hole wavefunctions within the confinement volume (*i.e.* the QD volume), thereby limiting the maximum attainable oscillator strength to unity overlap between the electron and hole wavefunctions. By contrast when the QD size is larger than *a*_*X*_, where the exciton size *L*_exciton_ is roughly given by the Bohr exciton radius (*L*_exciton_ = 2*a*_*X*_), the electron and hole motion is correlated by their mutual Coulomb attraction. In this regime, referred to as weak exciton confinement, the center of mass motion of the exciton becomes quantized and the relative motion between the electron and the hole can be assumed to be like the 1s hydrogen orbital, where the overall envelope function of the exciton can be expressed as [44],

(2) $$\begin{array}{c} F\left({\bar{r}}_{e},{\bar{r}}_{h}\right)=\frac{1}{\sqrt{N\left(a_{X}\right)}}e^{-\frac{\left|{\bar{r}}_{e}-{\bar{r}}_{h}\right|}{a_{X}}}f_{e}\left({\bar{r}}_{e}\right)f_{h}\left({\bar{r}}_{h}\right) \end{array}$$

(3) $$\begin{array}{c} F_{0}\left({\bar{r}}_{e},{\bar{r}}_{h}\right)=f_{e}\left({\bar{r}}_{e}\right)f_{h}\left({\bar{r}}_{h}\right) \end{array}$$

Critical is the additional factor $N\left(a_{X}\right)$ required to make sure that the exciton envelope function is normalized, and $N\left(a_{X}\right)<1$ is indicative of the electron and the hole motion being correlated in space, resulting in an increased electron–hole overlap and enhanced light matter coupling strength. It has been shown that this increased overlap results in an enhancement of the transition dipole moment [44], where the enhancement factor is $\frac{1}{\sqrt{N\left(a_{X}\right)}}$ , and corresponds to the annihilation of the exciton, resulting in a factor of $\frac{1}{N\left(a_{X}\right)}$ enhancement in the oscillator strength.

As discussed throughout this paper, the superradiant enhancement of light–matter interaction in MTSQDs comes from their large volume, and the ability to control their shape, and spatial compositional distribution, as shown in the atomic-resolution EDS map of an MTSQD in Figure 5(c). The overlaid markings in Figure 5(c) serve to capture the effect of the MTSQD’s large-volume, and uniform three-dimensional confinement, on the exciton. In such weakly confining QDs, the motion of the electron and the hole becomes correlated and thus the exciton is able to sample a larger number of Bloch unit cells within the confinement volume resulting in a collective enhancement of light–matter interaction in the QD. This is illustrated in Figure 5(c) with *L*_MTSQD_ > *L*_exciton_ and the exciton envelope function being able to sample different regions of the MTSQD confinement potential at a time. Also shown in Figure 5(c) is the fact that the coherent phase relation in the collective state shared by the exciton across the MTSQD volume comes from the exciton’s dipole (with its orientation defined by the MTSQD’s confinement potential), which leads to a giant transition dipole for the exciton and thus superradiant enhancement of light–matter interaction. Similar effects have also been observed in interface fluctuation GaAs QDs [10] and CsPbBr_3_ colloidal QDs [44] and are unique to such systems in which a three-dimensionally confined exciton is able to coherently sample a uniform confinement potential volume considerably larger than itself.

As a further validation of the enhancement of the oscillator strength owing to the superradiant effect in the MTSQDs, we employ equation (2) and solve for the electron and hole envelope functions under independent particle picture and under the effective mass approximation for the MTSQD size and shape guided by the STEM studies. We construct a 3D confinement volume of pyramidal shape with base diagonals (based on the known ∼{103} bounding facets) [24], [31], where we take the base size along [110] to be ∼30 nm, height to be ∼5 nm, and base size along [1−10] to be ∼30 nm, and employ a finite element method calculation to obtain $f_{e}\left({\bar{r}}_{e}\right)$ and $f_{h}\left({\bar{r}}_{h}\right)$ . We then include the electron–hole correlation ( $e^{-\frac{\left|{\bar{r}}_{e}-{\bar{r}}_{h}\right|}{a_{X}}}$ ) with the Bohr exciton radius ∼10 nm–15 nm (corresponding to Bohr exciton radius in bulk InGaAs) and numerically evaluate the oscillator strength enhancement factor $\frac{1}{N\left(a_{X}\right)}$ (For details, please see  Supplementary Material Section S1). We find the oscillator strength enhancement to be ∼2.5, consistent with the MTSQD oscillator strength (∼30) being a factor of ∼ 2.5–3 higher compared to the oscillator strength of typical InGaAs SAQDs that exhibit strong confinement. These results point to the uniqueness of the MTSQDs as a platform enabling control of light matter interaction by controlling the size and shape.

## 3 Conclusions

Beyond meeting all key figures-of-merit for technological advancement of photonic quantum information processing as a single photon source platform, both at the individual device level (brightness, purity, indistinguishability) and at the system level (scalability, spatial ordering, and spectral uniformity), MTSQDs are shown here to have controlled enhancement of light–matter interaction allowing for exploitation of the single photon superradiance phenomena. It is worth pointing out that MTSQDs are the only class of QDs that allows for tailoring the intrinsic oscillator strength and achieving reproducibly large *f* in scalable and spatially-ordered arrays. Such a unique characteristic would allow for future systematic superradiance studies, focusing on a combination of MTSQD’s intrinsic single-photon superradiance together with the phenomena of Dicke’s superradiance. This could be implemented by coupling multiple MTSQDs either vertically on a single mesa (as discussed in Section 2.1) or horizontally through collective excitation of the QDs through a shared electromagnetic mode (i.e. through photons emitted by the QDs embedded in a waveguide) [45], [46]. Furthermore, the SESRE approach is implementable in a wide array of material systems, spanning from lattice-matched to – mismatched materials, allowing the benefits from precise positioning and enhanced light–matter interactions to be used in different emission wavelength regimes.

The essence of MTSQDs’ unique structural and optical properties lies in their synthesis through the SESRE growth approach. Results discussed in this paper highlight that the single photon superradiant enhancement of light–matter interaction observed in MTSQDs originates from the reproducible controlled large-volume and shape of MTSQDs in arrays, enabled by the control of run-to-run growth conditions and control of nanomesa profile evolution during SESRE growth. It is also important to emphasize that SESRE driven spatially-selective growth does not require lattice mismatch induced strain to achieve spatially-selective growth. Indeed, the first SESRE quantum dots were realized in the lattice-matched GaAs/AlGaAs material system [23], [30].

## 4 Experimental methods

### 4.1 MTSQD array growth

The MTSQD samples studied in this paper were grown by molecular beam epitaxy (MBE) on GaAs (001) substrates patterned with pedestal nanomesas, arranged in arrays, sitting on top of a distributed Bragg reflector (DBR) mirror (as seen in Figure 2(a)). We note that the DBR mirror was designed with the purpose of enhancing only the photon collection efficiency of MTSQDs and our finite element method studies indicate that it contributes a factor less than 1.3 to Purcell enhancement [11]. The samples, grown under the same growth conditions, differ only in the size of the nanomesa arrays used for growth; with one sample having small (5 × 8) arrays (40 MTSQDs per array) and the other sample having large (50 × 50) arrays (2,500 MTSQDs per array), both with pitch 5 μm. Each sample used for growth consisted of a substrate of size ∼1 cm × ∼1 cm containing different smaller areas (∼1 mm × 1 mm) patterned with the nanomesa arrays. After growth the ∼1 cm × ∼1 cm substrate, now containing planarized MTSQD arrays, was cleaved into smaller pieces so that structural and optical characterization could be conducted on the different cleaved pieces from the same growth.

Before MTSQD growth, the GaAs (001) substrates containing the DBR structure [11] were patterned with arrays of square mesas of HSQ negative resist (∼70 nm thick), with HSQ mesa edges oriented along <100>, using electron-beam lithography (EBL, Raith EBPG 5150). The HSQ served as the mask for subsequent wet etching of the GaAs pedestal nanomesas. Etch rate of wet-etching solutions (NH_4_OH based) were carefully calibrated, allowing us to fabricate GaAs pedestal nanomesa arrays of with height ∼100 nm and lateral sizes ranging from ∼100 nm to ∼400 nm on both samples.

The growth structure (*i.e.* deposition layer sequence) and growth conditions were ensured to be the same for both samples by employing our MCTF approach [29]. The growth consists of: (i) deposition of 270 ML of GaAs, at temperature of ∼600 °C, *A*s_4_ flux of *P*_As4_ ∼ 2 × 10^−6^ Torr, and growth rate of 0.25 ML/s, which resulted in nanomesas developing a pyramidal morphology and an incumbent reduction of the mesa top size following SESRE (MTSQDs were targeted to form on nanomesas with starting lateral size of ∼300 nm, which after the 270 ML GaAs deposition had a top size of ∼30 nm). (ii) deposition of 4.25 ML of In_0.5_Ga_0.5_As, for MTSQD formation, at a temperature of ∼520 °C, *P*_As4_ ∼ 3 × 10^−6^ Torr, and growth rate of 0.5 ML/s. Right after InGaAs deposition, 20 ML of GaAs was deposited for pinching off the {103} sidewalls of the pyramidal nanomesas containing MTSQDs. (iii) deposition of ∼1250 ML of GaAs for planarizing the pyramidal nanomesa structures at ∼600 °C and As_4_ flux of P_As4_ ∼ 2 × 10^−6^ Torr. Thin AlAs layers (∼10 ML) were deposited in regular intervals during the GaAs layer depositions to act as marker layers for STEM characterization as seen in Figure 2(a)–(d). Apart from the array size, the only difference between the samples studied here was that the number of AlAs marker layer deposited on the sample containing (50 × 50) arrays was doubled (with respect to the (5 × 8) array sample) for better inspection of the nanomesa profile evolution during STEM studies.

### 4.2 TEM specimen preparation

Preparation of specimens for STEM examination, carried out using a dual-beam focused ion beam (FIB)/scanning electron microscope (SEM) instrument (Helios G4 UXe PFIB), consisted of creating electron-transparent lamella containing the MTSQDs through a modified version of the typically employed site-specific lift-out technique. The main modifications in our approach compared to typical protocols were in (i) use of electron beam induced deposition (EBID) of Pt marker structures on the substrate surface (underneath which MTSQDs were located), before trench-milling and lift-out, allowing for controlled FIB thinning of the lifted-out lamella containing MTSQDs, and (ii) backscattered electron (BSE) imaging of the AlAs marker layers on the lamella surface during FIB thinning to control the specimen thickness and assure that the MTSQDs are not milled away during thinning (*i.e.* MTSQDs are contained within the lamella). This approach leads to reproducible control of the position of the MTSQD within the electron-transparent lamella every time TEM specimens are prepared. The TEM specimens prepared for the studies in this paper were all done along the [110] cross-section.

### 4.3 STEM and EDS characterization

All STEM and EDS measurements presented here were conducted in a probe-corrected Spectra 200 X-CFEG STEM instrument. Atomic-resolution STEM imaging and EDS mapping conditions were chosen to maximize spatial resolution and thus were done with specimen oriented along the [1-10] zone axis and with a beam energy of 200 keV, beam current ∼100 pA, beam semi convergence angle of 25 mrad and probe aberrations corrected to 5th order, resulting in a spatial resolution of <0.8 Å (as determined from the fast Fourier transform (FFT) of the STEM images). STEM imaging was done using a high angle annular dark field (HAADF) detector set to inner and outer collection angles of ∼80 mrad–200 mrad for producing Z-contrast images arising from incoherent thermal diffuse scattering, minimizing diffraction-based contrast in the images. EDS data was acquired using a dual-X detector, with a solid-angle of collection of ∼1.8 sr. Off-zone axis EDS measurements were done with a ∼4° tilt away from the [1], [2], [3], [4], [5], [6], [7], [8], [9], [10] zone axis (along the [200] direction), to reduce the effects of channeling, and with a beam current of 500 pA to increase the counts from the X-ray characteristic peaks. The EDS elemental maps were calculated by extracting the integrated counts from X-ray characteristic peaks (*e.g.* In Lα1, Ga Kα1 and As Kα1) after removal of the Bremsstrahlung X-ray background counts.

### 4.4 Optical characterization

Large area photoluminescence (PL) imaging was done on the (50 × 50) array (see Figure 3(a)) using an in-house built tunable filter system [35]. The measurement allows for imaging emission of individual MTSQDs in the array over large areas (with the area probed being limited by the excitation area of the beam, ∼180 μm × 190 μm) and resolving their emission wavelength with a spectral resolution of ∼1.6 nm [35], where the emission intensity is normalized to ∼4.5 % of the saturation power for the brightest of emissions. Single photon measurements from individual MTSQDs (see TRPL data from Figure 4) were done using a resonant excitation scheme, in vertical excitation and vertical detection geometry, with a spatial resolution ∼1.2 μm and with the sample placed in a cryostat at 4 K temperature. The laser pulse width in the TRPL measurements was ∼3 ps, two orders of magnitude shorter than the MTSQD’s radiative decay lifetime, therefore allowing us to confidently probe the intrinsic exciton decay dynamics in the MTSQDs. For details on the resonant excitation measurement conditions, setup, and on the three-level model used for extracting radiative decay lifetimes from MTSQD neutral excitons please see ref. [11].

## Supplementary Material

Supplementary Material Details
