# Supplementary material for: Single photon superradiance enhanced light–matter interaction in spatially ordered shape and volume controlled single quantum dots: enabling on-chip photonic networks
Source: Nanophotonics. 2025 Sep 10;14(19):3157–68. doi: 10.1515/nanoph-2025-0270 (PMC12455285; doi:10.1515/nanoph-2025-0270)
Supplement: Supplementary file 1 — Supplementary Material Details [file j_nanoph-2025-0270_suppl_001.docx]

**Strictly adhere to the below given format and ensure the following:**

- **Make sure that the information given in the author’s statement below corresponds to the information in the manuscript, if applicable.**
- **If no information is provided, default statements will be set in place, as given below.**
- If **Acknowledgments** are not applicable, remove.
- If **Informed Consent** and **Ethical Approval** are not applicable, remove.

**Research funding** *(mandatory)*

This work is supported by the Air Force Office of Scientific Research grant number FA9550-22 1-0376, the Center for Nanoimaging (CNI) at the University of Southern California, and the Kenneth T. Norris Professorship.

**Author contributions** *(mandatory)*

All authors have accepted responsibility for the entire content of this manuscript and consented to its submission to the journal, reviewed all the results and approved the final version of the manuscript. LJ and QH grew the samples. LJ conducted the STEM and EDS characterization. QH, JZ and SL developed the optical instrumentation. QH conducted optical measurements. SC provided the theoretical analyses. AM coordinated and guided the overall project. All authors participated in manuscript writing.

**Conflict of interest** *(mandatory)*

The authors declare no competing interests.

**Data availability** *(mandatory)*

The datasets generated and/or analysed during the current study are available from the corresponding author upon reasonable request.
